# Cereal cystatins delay sprouting and nutrient loss in tubers of potato, *Solanum tuberosum*

**DOI:** 10.1186/s12870-015-0683-2

**Published:** 2015-12-21

**Authors:** Aurélie Munger, Marie-Aube Simon, Moustafa Khalf, Marie-Claire Goulet, Dominique Michaud

**Affiliations:** Centre de recherche et d’innovation sur les végétaux|Biotechnologie Université Laval, Québec, QC G1V 0A6 Canada; Present address: Services aux entreprises et formation continue, Cégep de St-Jérôme, St-Jérôme, J7Z 4 V2 QC Canada

**Keywords:** Cereal cystatins, Endogenous proteolysis, Potato (*Solanum tuberosum*), Transgenic crops, Tuber sprouting

## Abstract

**Background:**

Recent studies have reported agronomically useful ectopic effects for recombinant protease inhibitors expressed in leaves of transgenic plants, including improved tolerance to abiotic stress conditions and partial resistance to necrotrophic pathogens. Here we assessed the effects of these proteins on the post-dormancy sprouting of storage organs, using as a model potato tubers expressing cysteine protease inhibitors of the cystatin protein superfamily.

**Results:**

Sprout emergence and distribution, soluble proteins, starch and soluble sugars were monitored in tubers of cereal cystatin-expressing clones stored for several months at 4 °C. Cystatin expression had a strong repressing effect on sprout growth, associated with an apparent loss of apical dominance and an increased number of small buds at the skin surface. Soluble protein content remained high for up to 48 weeks in cystatin-expressing tubers compared to control (untransformed) tubers, likely explained by a significant stabilization of the major storage protein patatin, decreased hydrolysis of the endogenous protease inhibitor multicystatin and low cystatin-sensitive cysteine protease activity in tuber tissue. Starch content decreased after several months in cystatin-expressing tubers but remained higher than in control tubers, unlike sucrose showing a slower accumulation in the transgenics. Plantlet emergence, storage protein processing and height of growing plants showed similar time-course patterns for control and transgenic tubers, except for a systematic delay of 2 or 3 d in the latter group likely due to limited sprout size at sowing.

**Conclusions:**

Our data point overall to the onset of metabolic interference effects for cereal cystatins in sprouting potato tubers. They suggest, in practice, the potential of endogenous cysteine proteases as relevant targets for the development of potato varieties with longer storage capabilities.

**Electronic supplementary material:**

The online version of this article (doi:10.1186/s12870-015-0683-2) contains supplementary material, which is available to authorized users.

## Background

Dozens of papers have discussed the potential of plant protease inhibitors as effective antidigestive compounds to engineer herbivore pest resistance in food and commodity crops [1, 2]. Recent studies have also assessed their usefulness as ectopic modulators of endogenous proteases to introduce traits of agronomical value such as pathogen resistance or abiotic stress tolerance in leaf tissues [2], or to prevent unintended proteolysis of ectopically expressed biopharmaceuticals in plants used as vehicles for recombinant protein production [3]. Cysteine (Cys) protease inhibitors of the cystatin protein superfamily [4] were shown for instance to provide host plants with partial resistance to necrotrophic fungi [5] and broad tolerance to drought, chilling, oxidation or salt stress [6–8]. Recombinant cystatins were shown also to prevent degradation of recombinant proteins in leaf tissue when coexpressed as accessory proteins to inhibit endogenous proteolysis in the cytosol [9] or along the cell secretory pathway [10, 11]. Little is known about the endogenous targets of cystatins in planta, but the ectopic effects reported for these proteins, the large numbers of Cys protease-encoding genes in plant genomes [10] and the well established roles of Cys proteases in such key physiological processes as programmed cell death, senescence, defense and storage protein mobilization [12] now make the regulation of these enzymes an interesting route for crop improvement.

In this study we assessed the potential of recombinant cystatins to downregulate Cys protease activity and prevent protein loss in non-dormant storage organs, using cereal cystatin-expressing potato tubers as an example of economic value. Gene expression studies have established clear correlations between storage protein deposition or mobilization, cystatin content, and Cys protease activity in seeds or vegetative storage organs of different plants [4, 12]. For instance, a positive link was established between deposition of the major storage protein patatin, high transcript numbers for the 88-kDa Cys protease inhibitor potato multicystatin (PMC) and low Cys protease activity in developing potato tubers [13]. Patatin mobilization was shown by contrast to correlate with low numbers of PMC transcripts, Cys protease up-regulation and increased protease activity in sprouting tubers [14]. A simple working model was proposed to predict the fate of storage proteins in reproductive organs, involving the cystatin::Cys protease stoechiometric balance as a key determinant of the resulting output [4]. Inhibitory cystatins are actively synthesized in developing storage organs to eventually outnumber Cys proteases and promote storage protein deposition. An elevated, abscisic acid-dependent cystatin::Cys protease balance in dormant tissues allows for the pool of storage proteins to be maintained over dormancy and remain available to the growing plantlets upon germination or sprouting [13, 15–17]. A gibberellin-induced up-regulation of Cys protease genes concomitant with the repression of cystatin genes leads, finally, to a low cystatin::Cys protease balance favoring storage protein mobilization and plantlet growth [14, 17–20].

The physiological significance of a low cystatin::Cys protease balance upon germination was supported empirically with transgenic Arabidopsis lines overexpressing *AtCYS6*, a seed endogenous cystatin naturally responsive to gibberellins and abscisic acid [17]; or with Arabidopsis lines expressing *BrCYS1*, a heterologous cystatin from Chinese cabbage also responsive to these hormones [21]. In line with the assumed repressing effect of cystatins on germination, transgenic seeds constitutively expressing either cystatins exhibited low Cys protease activity and delayed germination compared to non-transgenic seeds [17, 21], in sharp contrast with *AtCYS6* knockout seeds showing higher Cys protease activity and early germination [17]. Here we assessed the impact of two ectopically expressed cereal seed cystatins, oryzacystatin I (OCI) [15] and corn cystatin II (CCII) [22], on the sprouting behaviour, protein catabolism and growing potential of potato tubers stored for several months at 4 °C.

## Results and discussion

### OCI accumulates at low levels in potato tubers

**Fig. 1:**
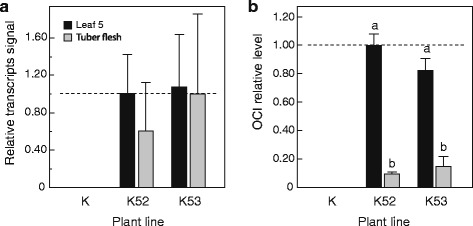
OCI expression in leaves and tubers of line K52, line K53 and control line K. **a** mRNA transcript relative levels in tuber flesh and leaf 5 down from the apex. **b** OCI relative content in tuber flesh and leaf 5 down from the apex. Data on panels (**a**) and (**b**) are expressed relative to mean levels in the fifth leaf of K52 plants (arbitrary value of 1.00, corresponding on panel (**b**) to ~0.85 % of total soluble proteins [24]). Each bar on both panels is the mean of biological replicates from three different plants ± sd. Bars with different letters on panel (**b**) are significantly different (post-anova Tukey’s test, *P* < 0.05)

Two OCI-expressing potato lines, K52 and K53, were selected for the experiments from a collection of independent transformants established earlier in our laboratory [23]. OCI in these lines bears no signal peptide at the N terminus and accumulates in the cytosol under the control of the Cauliflower mosaic virus (CaMV) 35S constitutive promoter. Reverse transcriptase (RT) polymerase chain reaction (PCR) assays were performed to estimate levels of OCI-encoding transcripts in leaves and tubers. Comparable levels of OCI transcripts were found in the 5^th^ leaf of lines K52 and K53, compared to undetectable levels in the 5^th^ leaf of control line K used as parent for genetic transformation (Fig. 1a). Roughly similar transcript levels were found in cDNA samples prepared from 1-cm^3^ tuber flesh pieces collected ~5 cm down from the apical buds of tubers stored for 36 weeks at 4 °C (Fig. 1a), indicating comparable ability of the viral promoter to drive OCI transgene expression in sprouting tubers and mature leaves.

Despite similar transcript signals, recombinant OCI was detected at low relative levels of ~0.08–0.15 % of total soluble proteins in tuber extracts of both lines K52 and K53, five to ten times less than levels measured in mature leaves [24] (Fig. 1b). One explanation for this could be a rapid turnover of the inhibitor in heterologous cellular environments harbouring distinct proteolytic machineries, as discussed earlier for a number of recombinant proteins reported to be unstable in plant-based expression systems [3]. A more likely explanation given the documented stability of plant cystatins in protease-rich heterologous environments such as plant leaf and microbial cells [10, 11, 25] would be a natural sink effect of highly abundant storage proteins on amino acid resources, inherently unfavorable to heterologous protein accumulation [26]. Studies have reported higher levels of recombinant protein in reproductive organs of storage protein-depleted mutants [27, 28] or transformants [29–32], presumably associated with increased energy and/or amino acid resources in planta. Such observations, while strengthening the idea of a limited proteome plasticity in storage organs, could also explain the low levels of recombinant proteins such as OCI (this study) or tomato cathepsin D inhibitor in transgenic potato tubers [26] compared to steady-state levels in leaves of the same plants.

### OCI expression delays tuber sprouting at 4 °C

**Fig. 2:**
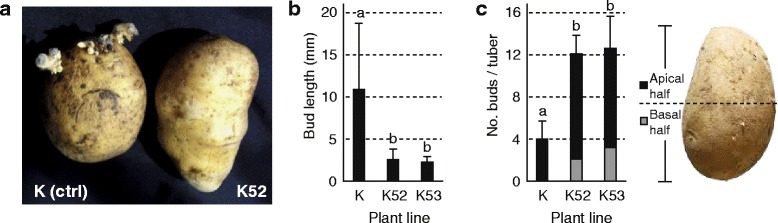
Number, length and distribution of buds and sprouts on tubers of lines K, K52 and K53 stored for 48 weeks at 4 °C. **a** General aspect of K and K52 tubers after storage, showing well developed sprouts and visible skin deterioration on tubers of the control line. **b** Mean length of buds and sprouts at the surface of stored tubers. **c** Number and distribution of buds and sprouts on the apical (*black bars*) and basal (*grey bars*) halves of stored tubers. Each bar on panels (**b**) and (**c**) is the mean of six biological (tuber) replicate values ± sd. On each panel, bars with a different letter are significantly different (post-anova Tukey’s test, *P* < 0.05)

Distribution patterns of growing buds and sprouts were recorded on stored tubers of line K52, line K53 and control line K to look for eventual macroscopic effects of OCI on tuber sprouting despite a limited accumulation in tuber flesh tissue (Fig. 2). At least six ~10 cm-long tubers harvested from different plants of each line were stored in the dark at 4 °C for 48 weeks (*ca.* 11 months) prior to visual inspection. Following a resting period of several weeks after harvest, endodormancy is released in potato tubers and one, or a few, apical buds start growing and using storage nutrients [33]. Accordingly, three to five well developed, ~10 mm-long sprouts were found at the apex of stored control tubers after 48 weeks, associated with visible signs of skin dehydration (Fig. 2). By comparison, about 12 small, ~2.5 mm-long buds were counted on tubers of lines K52 and K53 (Fig. 2b, c), associated with a visually unaltered skin surface similar to that of freshly harvested tubers (see Fig. 2a for line K52). Similar observations were made with a number of additional lines expressing OCI at comparable or lower levels (Additional file 1), suggesting a sprout-repressing effect of this protein even at very low levels in tuber tissue.

As expected given the establishment of apical dominance at early sprouting [34], most, if not all, growing sprouts on control line K tubers were found at-or around-the apex, on the upper (apical) half of the tuber (Fig. 2c). Buds on K52 and K53 tubers were also found mostly on the apical half, but a consistent number of two or three buds were also observed on the basal half (Fig. 2c). These observations pointed, overall, to an alteration of the apical dominance pattern in OCI-expressing tubers and to a significant retarding effect of recombinant OCI on sprout growth presumably associated with limited metabolic activity in the tubers and/or apical buds. These data suggest, in practice, the feasibility of preventing early tuber sprouting during long-term storage by the ectopic expression of recombinant cystatins at low levels, with a minimal demand in endogenous amino acid resources and a likely negligible effect on the tuber protein complement.

### Storage protein catabolism is delayed in OCI-expressing tubers

**Fig. 3:**
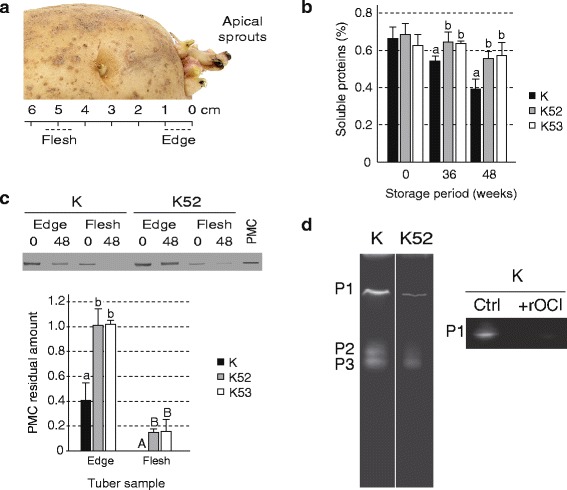
Soluble proteins, potato multicystatin (PMC) and protease activity in tubers of line K52, line K53 and control line K stored for different periods at 4 °C. **a** Diagram showing the position of tuber ‘edge’ and ‘flesh’ tissues collected for the analyses. **b** Total soluble proteins in crude protein extracts of flesh tissue after storage for 0, 36 or 48 weeks. Data are expressed as percentages on a fresh weight basis. **c** Immunodetection of PMC in crude protein extracts of tuber edge and flesh tissues upon harvesting (0 week) or after storage for 48 weeks. Densitometric data are expressed for stored tubers relative to initial levels at time 0 (arbitrary value of 1.0). PMC on the immunoblot image corresponds to PMC purified from tuber skin. **d** Gelatin hydrolysis zymogram for the main proteases of flesh tissue (P1, P2 and P3) in line K and line K52 tubers after storage for 48 weeks. +rOCI, crude extract incubated with recombinant OCI expressed in *E. coli* before electrophoretic migration. Each bar on panels (**b**) and (**c**) is the mean of six biological (tuber) replicates ± se. Identical volumes of crude extract were loaded in each well on panels (**c**) and (**d**). For each time point or tissue type, bars with the same letter are not significantly different (post-anova Tukey’s test ; *P* > 0.05)

Soluble proteins were assayed in tubers of lines K, K52 and K53 to assess the overall impact of OCI expression on storage protein deposition in developing tubers and protein catabolism during early sprouting, after storage for 36 or 48 weeks at 4 °C (Fig. 3). One-cm^3^ tuber tissue pieces were collected at 0 cm (‘edge’ tissue containing skin cells) or 4.5 cm (‘flesh’ tissue) down from the apex, beneath the emerging apical sprouts (Fig. 3a). Soluble protein content in flesh tissue samples of freshly harvested tubers (time 0) was estimated at ~0.65 % fresh weight in both transgenic and control lines, suggesting a null impact of OCI on total storage protein deposition in growing tubers (anova; *P* = 0.398, F = 1.077) (Fig. 3b). By contrast, protein content in flesh tissue of line K decreased to 0.55 % fresh weight after 36 weeks and then to 0.39 % after 48 weeks, while remaining significantly higher in flesh tissue of K52 and K53 tubers after both 36 (*P* = 0.014, F = 9.447) and 48 weeks (*P* = 0.010, F = 11.13), at ~0.55–0.62 % fresh weight.

Immunodetections were carried out to compare PMC levels in control and transgenic line protein samples. PMC accumulates in growing potato tubers along with the major storage protein patatin, and then disappears upon sprouting to release free endogenous Cys proteases and help provide free amino acids for plantlet growth [13, 14]. Similar to total soluble proteins, PMC was found at comparable levels in OCI-expressing and control tubers at time 0 in both edge and flesh tissues (see immunoblot image on Fig. 3c), again suggesting no effect of OCI on tuber protein deposition. Extensive degradation of the endogenous cystatin was observed in line K after 48 weeks, down to a residual level of 40 % compared to time 0 in edge tissue, and to barely detectable levels in flesh tissue. By comparison, PMC content in edge tissue remained unchanged in both lines K52 and K53 after 48 weeks (anova; *P* > 0.05), and was still detectable in flesh tissue despite significant degradation (Fig. 3c).

Gelatin-polyacrylamide gel electrophoresis (PAGE) zymograms [35] were produced to visualize major protease (gelatinase) forms in stored tuber protein samples (Fig. 3d). In accordance with the anti-papain inhibitory activity of OCI [15] and the predominance of papain-like Cys proteases in sprouting tubers [36], protease activity after 48 weeks was less important in OCI-containing protein samples than in line K samples. rOCI, a recombinant form of OCI produced in *E. coli* [36], was incubated with line K samples prior to gelatin-PAGE to confirm the occurrence of OCI-sensitive Cys protease(s) in sprouting tubers (Fig. 3d). As shown for the major gelatinase P1, rOCI had a sharp inhibitory effect on endogenous proteases causing an almost complete loss of gelatinase activity compared to the non-inhibited control. These findings, although not excluding alternative effects in vivo, support the hypothesis of a protease inhibition-mediated mechanism for the sprouting repression effect of OCI in potato tubers, in line with the protease regulatory role of this inhibitor in rice seeds [15] and as also suggested for the germination-retarding effect of recombinant cystatins in Arabidopsis seeds [17, 21].

### Starch processing is delayed in OCI-expressing tubers

Sugar assays were conducted to assess the impact of OCI expression on starch processing and soluble sugar content in transgenic tubers (Table 1). Starch hydrolysis is known to occur in sprouting tubers, typically associated with an increase in soluble sugars [37]. Starch content in flesh samples of freshly harvested tubers was here estimated at ~14.5 % of total fresh weight for the three tested lines (anova; *P* = 0.594, F = 0.569), comparable to starch levels observed earlier in tubers of the same cultivar [26, 38]. By contrast, starch content in line K decreased to 11.8 % after 36 weeks of storage, compared to a greater, almost unchanged mean value of ~14 % in the OCI-expressing tubers (*P* = 0.002, F = 19.40). Starch content further decreased in control tubers after 36 weeks to reach 6.3 % of total fresh weight at 48 weeks, lower than the levels of about 8 % measured in OCI-expressing tubers (*P* = 0.012, F = 10.12).

**Table 1:** Starch, sucrose and glucose contents in control and OCI-expressing potato tubers stored at 4 °C for 0, 36 or 48 weeks

Sugar	Concentration (% fresh weight) ^1^								
	0 week (harvest)			36 weeks			48 weeks		
	K	K52	K53	K	K52	K53	K	K52	K53
Starch	14.8	14.6	14.3	11.8 a	14.1 b	13.8 b	6.28 A	7.87 B	8.40 B
Sucrose	0.20	0.19	0.20	0.45	0.29	0.30	1.33 A	0.57 B	0.71 B
Glucose	0.16	0.13	0.12	0.28	0.24	0.29	1.03	1.00	0.87

^1^Data are expressed as mean relative contents in flesh tissue of three tubers harvested from different plants. For each storage period, data with different letters on the same line are significantly different (post-anova Tukey’s test, *P* < 0.05)

Similar to starch, comparable levels of sucrose (anova; *P* = 0.969, F = 0.032) and glucose (*P* = 0.304, F = 1.463) were found in tubers of control and transgenic lines upon harvesting, estimated at ~0.20 and ~0.14 % of tuber fresh weight, respectively, similar to previously reported contents [26, 38]. Glucose content gradually increased in tubers of all three lines, to reach about 1 % of tuber fresh weight at 48 weeks (*P* = 0.736, F = 0.323). Sucrose content also increased during storage but remained lower in the OCI tubers, about half the levels observed in control tubers after storage for 48 weeks (*P* = 0.002, F = 22.40). These observations suggest on the one hand a negligible effect of OCI expression on the deposition of starch and soluble sugars in growing tubers. They indicate on the other hand a significant interfering effect of the cystatin on sugar catabolism during long-term storage, concomitant with the above-described repressing effects of this protein on sprouting and endogenous protease activity.

### Germination and plantlet growth are delayed, but not compromised, in OCI-expressing tubers

**Fig. 4:**
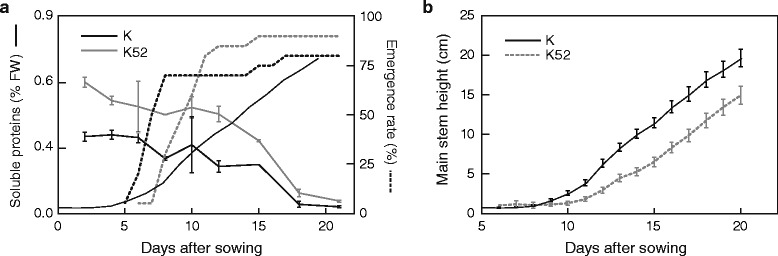
Growth parameters of plants emerged from line K52 and control line K tubers stored for 48 weeks at 4 °C. **a** Plantlet emergence rate and total soluble protein content in sown tubers, 0 to 21 d post-sowing. Protein contents, reported as percentages of tuber fresh weight (FW) [26], represent mean values for three biological (tuber) replicates ± se. Emergence rates are expressed as cumulative percentage of tubers giving emerged shoots, out of 20 tubers sown (100 %). **b** Main stem height of emerged plants, 5 to 20 d post-sowing. Each point is the mean of six biological (plantlet) replicates from different tubers ± se

A sowing assay was conducted with tubers of lines K and K52 stored for 48 weeks at 4 °C to assess the ability of OCI-expressing tubers of producing viable plantlets despite limited bud outgrowth after long-term storage, and hence of being of eventual interest to produce seed tubers for vegetative propagation (Fig. 4). In brief, plantlets started to emerge from control tubers 5 d after sowing to reach an overall emergence rate of 80 % (i.e. 16 tubers emerged out of 20 sown) after 17 d, comparable to line K52 tubers exhibiting a 90 % emergence rate (18 tubers out of 20) recorded from 6 to 15 d post-sowing (Fig. 4a). Soluble protein content showed a gradual decrease after sowing in flesh tissue of both control and K52 tubers, down to a low, negligible residual level after 18 d (Fig. 4a). Overall plantlet growth –as estimated by mean plant height after emergence– showed a similar trend over time for the two lines, let apart a systematic delay of 2–3 d for the OCI-expressing tubers (Fig. 4b). Likewise, total cumulative numbers of leaves and stems on emerged plantlets showed similar incremental patterns for the two lines, except for a 2–3 d delay with the K52 line (data not shown). These observations suggest overall no clear effect of recombinant OCI on plantlet emergence and growth, with the notable exception of a short delay for the transgenic tubers likely explained by limited sprout size after storage.

### Corn cystatin II also delays storage protein breakdown in potato tubers

**Fig. 5:**
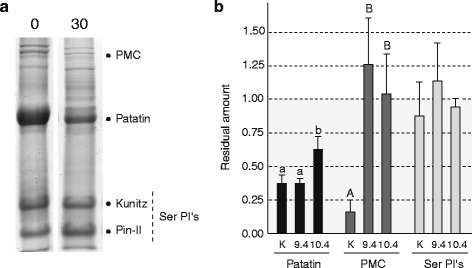
Relative amounts of patatin, PMC and Ser protease inhibitors (PI’s) in tuber flesh of control line K and corn cystatin II (CCII)-expressing lines 9.4 and 10.4 stored for 0 or 30 weeks at 4 °C. **a** SDS-PAGE protein profile of total soluble proteins in tubers of line K after storage for 0 or 30 weeks. **b** Residual amounts of patatin, PMC or Ser PI’s after 30 weeks compared to initial amounts at time 0 (arbitrary value of 1.00). Similar amounts of tuber crude extract were loaded in each well. Each bar on panel (**b**) is the mean of three biological (tuber replicate) values ± sd. For each protein, bars with a different letter are significantly different (post-anova Tukey’s test, *P* < 0.05). See ref. [26] for MS/MS identification of the tuber test proteins following electrophoresis

Patatin, PMC and the serine (Ser) protease inhibitors Kunitz trypsin inhibitors and proteinase inhibitor II [26] were monitored in transgenic potato tubers engineered to express a maize functional homologue of OCI, corn cystatin CCII [22], to gain further confirmation for the retarding effect of ectopic cystatin expression on storage protein catabolism (Fig. 5). Two CCII-expressing potato lines, line 9.4 and line 10.4, were selected for the experiments among a collection of stable transformants derived from control parental line K [5]. Similar to OCI in lines K52 and K53 (see above), CCII in lines 9.4 and 10.4 bears no N-terminal signal peptide and accumulates in the cytosol under the control of the CaMV 35S constitutive promoter. Ten cm-long tubers produced from greenhouse-acclimated plantlets were harvested for each line and stored at 4 °C for 0 (‘harvest’ control) or 30 weeks (*ca.* 7 months) prior to storage protein analysis. Soluble proteins extracted from flesh tissue were resolved by sodium dodecyl sulfate (SDS)-PAGE and stained with Coomassie blue (Fig. 5a), and the relative amounts of patatin, PMC and Ser protease inhibitors estimated by densitometric analysis of the corresponding bands (Fig. 5b). Unlike the Ser protease inhibitors still found at their initial levels upon harvest, patatin and PMC underwent extensive degradation in tubers of control line K after 30 weeks, down to residual levels of ~35 and ~15 % the amounts found in freshly harvested tubers, respectively. By contrast, patatin and PMC showed respective residual levels of up to 60 % and about 100 % after the same period in CCII-expressing tubers, well above the corresponding levels in control tubers (anova; *P* < 0.05). These observations indicate overall a significant delaying effect of CCII on storage protein catabolism in stored tubers, as observed above with OCI in lines K52 and K53.

## Conclusions

Previous studies reported ectopic effects for recombinant protease inhibitors in transgenic plants, altering such important processes as programmed cell death, leaf senescence, protein biosynthesis, protein turnover and stress-inducible protein expression [5, 39–42]. Usually considered as pesticidal compounds to inhibit herbivorous pest digestive proteases [2], these proteins are now seen also as potential regulators of endogenous proteolysis in planta for the implementation of valuable quality traits such as salt and drought tolerance [8, 43, 44], chilling or oxidative stress tolerance [6, 41], resistance to microbial pathogens [5], tolerance to nitrogen deficiency [45], increased protein content [41] or reduced endogenous protease activity in plant protein biofactories [9–11]. Here we document the retarding effects of these proteins on storage protein catabolism and bud outgrowth in a vegetative reproductive organ. Cystatin ectopic expression was shown previously to delay seed germination in Arabidopsis, presumably via an inhibition of endogenous Cys proteases involved in storage protein breakdown [17, 21]. We report similar effects, and the practical potential of such effects, for cereal cystatins ectopically expressed in vegetative storage organs, using potato tubers as a model of economic value.

Potato is an important staple crop worldwide and sustained efforts have been made over the years to improve its attributes as a food, using both classical breeding and biotechnological approaches [46]. The potato tuber is a rich source of valuable nutrients, such as starch and proteins [47], that need to be preserved between harvesting and eventual human consumption or use as ‘seed’ material for vegetative propagation [48]. Towards this goal, our data provide empirical evidence for the potential of recombinant cystatin expression as an ‘in-built’ strategy to control sprouting and nutrient depletion during storage, in complement to current approaches involving chemical sprouting inhibitors [49–51] or physical means such as postharvest irradiation or pressure treatments [52, 53]. Work is underway to characterize interactions between endogenous Cys proteases and recombinant cystatins in cystatin-expressing tubers, taking into account the complex hormone- and sugar-mediated signaling networks shaping the dormancy and sprouting processes of potato tubers [54, 55]. Work is also underway to compare the anti-sprouting effects of OCI and CCII with the eventual effects of improved cystatin functional variants developed earlier in our laboratory [56].

## Methods

### Plant lines

Transgenic potato lines (*Solanum tuberosum* cv. Kennebec) expressing OCI or CCII were selected from in-house collections of independent transformants maintained in vitro at 20 °C in 25 mm-wide x 15-cm long glass tubes, under a 16 h:8 h day/night photoperiod [23, 57]. Gene constructs for transformation included either the OCI-encoding sequence (GenBank Accession No. J03469) or a CCII-encoding sequence (GenBank Accession No. D38130) without the native N-terminal peptide signals for cellular secretion. They also included an original CaMV 35S promoter (OCI lines) or a duplicated version of this promoter (CCII lines); a tobacco etch virus enhancer sequence (CCII lines); and a nopaline synthase (OCI lines) or a CaMV 35S (CCII lines) terminator sequence (see refs. [23] and [57] for details on gene constructs). Line K used as parental line for transformation was taken as a control for comparative purposes [57].

### Tubers

Tubers were produced from in vitro-grown plantlets acclimated in greenhouse for 2 weeks under a 16 h/8 h light–day photoperiod, and then let to grow for 20 weeks in 25-cm (10-inch) wide pots filled with vermiculite. Three to six tubers of similar size (*ca.* 10 cm-long) were harvested from different plants and used for cystatin expression monitoring, sprout inspection, sowing assays or compositional analyses after storage in the dark at 4 °C for 0, 30, 36 and/or 48 weeks. Tuber samples for cystatin monitoring and compositional analyses were ground to a fine powder in liquid nitrogen, and the resulting powder lyophilized to dryness and stored at −80 °C until use.

### OCI expression

OCI transcripts were monitored by RT PCR using total RNA samples and the following oligonucleotide primers: 5’-GAACGACCTCCACCTCGTCGACCTC and 3’-GTACAAAGTGCCAGCGACAACTTGCT. Total RNA was extracted from tuber tissue powder (see above) or from the fifth leaf of ~30 cm-tall plants (down from the apex [24]), using the Concert Plant RNA Reagent™ kit (Life Technologies, Burlington ON, Canada). After precipitation in isopropanol, total RNA was washed in 75 % (v/v) ethanol, dissolved in RNAse-free water, and assessed for quantity and quality by the monitoring of A_260_/A_280_ and A_260_/A_230_ absorbance ratios with a NanoDrop™ 1000 spectrophotometer (Thermo Fisher Scientific, Mississauga ON, Canada) according to manufacturer’s instructions. cDNA populations were synthesized using the Superscript kit for cDNA synthesis (Life Technologies) and used as templates for PCR. The PCR amplicons were resolved by 1 % (w/v) agarose gel electrophoresis and visualized by ethidium bromide staining. For quantitation, the gels were digitalized with an Amersham Image Scanner (GE Healthcare, Baie d’Urfé QC, Canada) prior to computer processing and image analysis using the Phoretix 2D Expression software, v. 2005 (NonLinear Dynamics, Durham NC, USA) [42]. At least three tuber or leaf replicates were used for each line to allow for statistical assessment of the data (anova; α = 0.05).

### OCI content

OCI in leaf and tuber extracts was titrated using the colorimetric protein substrate azocasein and E-64-calibrated papain (EC 3.4.22.2) as a target protease [58]. Soluble proteins were extracted from tuber [or leaf] powder (see above) in 100 mM sodium phosphate, pH 6.5, containing 1 mM EDTA. Papain activity was monitored in 100 mM sodium phosphate buffer, pH 6.5, containing 1 mM EDTA, 1 mM phenylmethylsulfonyl fluoride, 5 mM l-cysteine and 0.1 % (v/v) Triton X-100 (Sigma-Aldrich, Mississauga ON, Canada). An arbitrary value of 100 % residual activity (0 % inhibition) was assigned to papain activities in leaf or tuber extracts of control line K.

### Soluble proteins and sugars

Soluble proteins were assayed according to Bradford [59] with chicken egg white albumin as a standard, after extracting proteins from lyophilized tuber powder as described above. Soluble sugars in powder samples were extracted in 80 % (v/v) ethanol for 30 min at 80 °C. After centrifugation for 5 min at 3000 g, the supernatant was recovered for glucose and sucrose enzymatic quantitation in the presence of ATP and NAD [60]. The pellet was resuspended in 0.2 M KOH for starch quantitation. After incubation for 30 min at 100 °C, the pH was adjusted to 5.5 with 1 M acetic acid. The starch fraction was hydrolyzed with amylase (E.C.3.2.1.1) and amyloglucosidase (E.C.3.2.1.3), and assayed by enzymatic determination of released glucose [60].

### PMC, storage proteins and endogenous proteases

PMC, patatin, Kunitz inhibitors and potato proteinase II in tuber protein extracts were resolved by 10 % (w/v) SDS-PAGE, and their relative content determined by densitometry of Coomassie blue-stained gels as described above for the OCI amplicons. PMC was also quantified on nitrocellulose sheets following immunoblotting, after detection with polyclonal anti-PMC primary antibodies. Protease activities in sprouting tubers were visualized by mildly denaturing gelatin/SDS-PAGE [35], with or without prior incubation of the protein extracts with rOCI, a recombinant form of OCI expressed in *E. coli* using the glutathione *S*-transferase system [36].

### Sowing bioassay

A sowing (‘germination’) assay was conducted with tubers of line K52 and control line K to detect eventual macroscopic effects of OCI expression on growth and development of the emerging plantlets. Twenty ~10 cm-long tubers stored at 4 °C for 48 weeks (see Fig. 1a) were sown in vermiculite-containing, 25-cm (10-inch) wide pots, and the plantlets were left to grow for three weeks in greenhouse. Sprouting emergence rates and stem height of the growing plants were monitored daily over 20 days. About 30 additional tubers of each line were sown in parallel, and three of them collected daily, to determine total soluble protein content in flesh tissue over the same period. Tuber processing and protein determinations were performed as described above for freshly harvested and stored tubers.

## Additional file

Additional file 1:
**Mean length of buds and sprouts on stored tubers of transgenic potato lines expressing OCI at different levels.** (PDF 24 kb)

## Abbreviations

CCII: corn cystatin II
Cys: cysteine
OCI: oryzacystatin
PAGE: polyacrylamide gel electrophoresis
PCR: polymerase chain reaction
PMC: potato multicystatin
RT: reverse transcriptase
SDS: sodium docedyl sulfate
Ser: serine
