# Supplementary material for: Cereal cystatins delay sprouting and nutrient loss in tubers of potato, Solanum tuberosum
Source: BMC Plant Biol. 2015 Dec 21;15:296. doi: 10.1186/s12870-015-0683-2 (PMC4687224; doi:10.1186/s12870-015-0683-2)
Supplement: Additional file 1: — Mean length of buds and sprouts on stored tubers of transgenic potato lines expressing OCI at different levels. (PDF 24 kb) [file 12870_2015_683_MOESM1_ESM.pdf]

Additional file 1 : Mean length of buds and sprouts on stored tubers of transgenic potato lines expressing OCI at different levels [1]

OCI line	OCI content (% TSP) [2]	Bud/sprout length (mm) [3]
K2	0.023 ± 0.007	3.6 ± 1.0
K3	0.033 ± 0.007	3.9 ± 0.7
K4	0.008 ± 0.004	4.5 ± 0.4
K5	0.146 ± 0.010	2.4 ± 0.4
K7	0.046 ± 0.012	2.8 ± 0.3
K21	0.072 ± 0.012	5.1 ± 1.1
K24	0.018 ± 0.009	4.5 ± 1.2

[1] These lines were produced as reported earlier [23], OCI content determined as described for lines K52 and K53 (see Methods), and stored tuber buds/sprouts examined after storage in the dark for 48 weeks at 4˚C.

[2] OCI content is expressed relative to total soluble proteins (TSP) in flesh tissue (%). Each value is the mean of three biological replicates from tubers of different plants ± SD.

[3] Data are mean length of buds and sprouts at the skin surface of stored tubers. Each value is the mean of six biological (tuber) replicate values ± SD.
